# Single-center early efficacy evaluation of Fontus branch-type intraoperative stenting for acute type a aortic dissection

**DOI:** 10.1186/s13019-025-03710-5

**Published:** 2025-12-27

**Authors:** Chong Sheng, Yuanxi Luo, Han Feng, Qing Zhou

**Affiliations:** 1 Department of Cardiovascular Surgery, Affiliated Hospital of Medical School, Nanjing Drum Tower Hospital, Nanjing University, 321 Zhongshan Road, Jiangsu, Nanjing, 210000 China; 2 Department of Cardiovascular Surgery, Nanjing Drum Tower Hospital, Chinese Academy of Medical Sciences & Peking Union Medical College, Peking Union Medical College Graduate School, Nanjing, China

**Keywords:** Acute type a aortic dissection, Fontus stent, Cronus stent

## Abstract

**Objective:**

To explore the clinical application value of the new Fontus intraoperative single-branch stent in acute type A aortic dissection (ATAAD).

**Methods:**

Ninety ATAAD patients admitted to the Department of Cardiothoracic Surgery of Nanjing Gulou Hospital from March 2022 to June 2023 who met the exclusion criteria were retrospectively analyzed, with Fontus stent applied intraoperatively in 33 cases and Cronus stent applied intraoperatively in 57 cases. Clinical data of preoperative baseline data, intraoperative indexes, perioperative period and postoperative follow-up were collected from both groups.

**Results:**

The rate of false lumen closure of the descending aorta at the level of the pulmonary bifurcation was significantly higher in the Fontus group than in the Cronus group; and the rate of endoleak was lower after Fontus stent application for modified arch island anastomosis.

**Conclusions:**

The new Fontus intraoperative single-branch stent is safe and effective in ATAAD and can reduce the incidence of postoperative endoleak.

## Introduction

Acute type A Aortic dissection (ATAAD) is a life-threatening condition with rapid onset, acute disease, high mortality, and poor prognosis without surgical management, requiring prompt diagnosis and rapid and effective surgical treatment [1]. It requires timely diagnosis and rapid and effective surgical treatment. Currently, there are several strategies for the arch management of type A aortic dissection with aortic arch involvement: partial aortic arch replacement, total aortic arch replacement with stenting elephant trunk surgery (Total Arch Replacement + Stented Elephant Trunk), island anastomosis (en bloc technique), intraoperative stenting, and so on [2, 3]. Total Arch Replacement + Stented Elephant Trunk is the most commonly used procedure in China. Total Arch Replacement + Stented Elephant Trunk is currently the most commonly used arch management in China, whereas the rest of the techniques simplify arch manipulation to a certain extent and all achieve good early and midterm results.The Cronus intraoperative stent, originally developed by Prof. Lizhong Sun for ATAAD in 2003, is easier to place in the descending aorta than the conventional soft elephant trunk, but its prosthesis is uncoated, prone to blood leakage, and the false lumen of the distal descending aorta is not easily closed [4]. The Fontus branch-type intraoperative stent (produced by MicroPort Endovascular MedTech (Shanghai) Co., Ltd., launched in 2021, material: nitinol covered stent and polyester graft) avoids the anastomosis of the left subclavian artery, which simplifies the surgical operation and has the advantages of reducing blood seepage and increasing the rate of distal false lumen closure. This study compared the Fontus stent with the Cronus stent, which has been widely used in our center, to evaluate its early efficacy as an upgraded product. We retrospectively analyzed the clinical data of patients with acute Stanford type A aortic dissection treated with Fontus stent or Cronus stent in the Department of Cardiothoracic Surgery of Nanjing Gulou Hospital from March 2022 to June 2023, and the treatment effects are reported as follows.

## Objects and methods

### Study subjects

Retrospectively analyzed 90 patients with acute type A aortic dissection who were treated with Fontus stent or Cronus stent in the Department of Cardiothoracic Surgery of Nanjing Gulou Hospital from March 2022 to June 2023, of which 33 patients were treated with Fontus stent (Fontus group), and 57 patients were treated with Cronus stent (In the Fontus group, there were 25 males and 8 females, aged 30–74 years, with a mean of (51.7 ± 14.2) years, and in the Cronus group, there were 48 males and 9 females, aged 23–81 years, with a mean of (50.9 ± 12.4) years. The retrospective analysis was approved by the Ethics Committee of Nanjing Drum Tower Hospital (reference number: 2020-185−01, approved in 2020 for this type of study protocol; data collection and analysis for this study commenced in 2022) and patients gave informed consent whenever possible.

### Exclusion criteria

(1) entrapment confined to the ascending and/or arch; (2) severe preoperative malperfusion syndrome; (3) vertebral artery variant; (4) entrapment due to infection, uncontrolled severe infection and associated sepsis, shock, or multiorgan failure, and inability to tolerate anesthesia and extracorporeal circulation; (5) major surgical procedures within 3 months; (6) history of active bleeding, clotting disorder or refusal of blood transfusion; (7) allergy to materials such as contrast media, nickel-titanium alloys, coated artificial blood vessels; (8) life expectancy of less than 12 months.

### Surgical methods

The chest is opened in the middle of general anesthesia, and extracorporeal circulation is usually established by cannulation in the femoral artery, right axillary artery and right atrium. When the nasopharyngeal temperature is lowered to 25℃~30℃, the aorta is blocked, and the ascending aorta is incised for collateral myocardial perfusion with cold blood stopping fluid. Aortic root management is determined by root techniques such as ascending aortic replacement, Bentall, and aortic valvuloplasty, primarily based on leaflet and sinus morphology. If necessary, simultaneous coronary artery bypass grafting was performed. Total arch replacement and stenting of the distal descending aortic prosthesis is performed in the arch, which requires deep hypothermic circulatory arrest (DHCA) with downstream selective cerebral perfusion or distal open anastomosis with bilateral cerebral perfusion.The appropriate type of Fontus stent or Cronus stent was selected based on the diameter of the true lumen of the descending aorta and the diameter of the left subclavian artery measured on preoperative aortic CTA. The Fontus stent diameter was typically 0–10% larger than the true lumen diameter, and the length typically covered the proximal descending aorta down to the T6-T8 level. Cronus stent sizing followed a similar principle. The stent was implanted openly into the true lumen of the descending aorta through the aortic arch incision. Follow-up was performed by outpatient review and telephone. Aortic CTA was periodically reviewed at 3 months and annually after the procedure to assess recovery.

### Observation indexes and follow-up

Record and comparatively analyze the operation time, extracorporeal circulation time, aortic block time, deep hypothermia stopping circulation time, operation mode, postoperative morbidity and mortality rate and complications, ventilator-assisted time, 24 h drainage flow, blood product dosage, ICU stay time, postoperative hospitalization time, mortality rate at follow-up, stent internal leakage rate, and the situation of the distal pseudo-lumen closing rate of the stent in the two groups of patients.Aortic CTA was periodically reviewed postoperatively (at 3 months, 1 year, and annually thereafter) to assess aortic remodeling.

### Statistical methods

SPSS18.0 software was applied for the statistical analysis of the result data. Normally distributed count data were expressed by‾ x ± s, and the comparison of the data before and after surgery was performed by the independent samples t-test; the measurement data were expressed by using the frequency and percentage (%), and were analyzed by using the x^2^ test. *p* < 0.05 was considered the difference was statistically significant.

## Results

### Preoperative data

Comparison of the two groups of patients at the time of admission in terms of age, gender, preoperative aortic valve lesions, degree of involvement of entrapment, comorbidities and other preoperative clinical data, the difference was not statistically significant (*P* > 0.05). See Table 1.

**Table 1:** Comparison of preoperative general clinical data of patients in Fontus group and Cronus group

Sports event	Fontus group (*n*=33)	Cronus group (*n*=57)	*P*-value
Male (cases, %)	25 (75.8)	48 (84.2)	0.23
Age ($\overline{x}$±s, years)	51.7±14.2	50.9±12.4	0.77
BMI ($\overline{x}$±s, kg/m^2^)	24.9±4.1	26.9±4.6	0.10
Comorbidities (cases, %)			
Coronary heart disease	0 (0.0)	2 (3.5)	0.40
High blood pressure	22 (66.7)	45 (78.9)	0.15
Diabetes	0 (0.0)	2 (3.5)	0.40
Chronic obstructive pulmonary disease (COPD)	0 (0.0)	1 (1.8)	0.63
Renal insufficiency	1 (3.0)	2 (3.5)	0.70
Cerebral hemorrhage	1 (3.0)	4 (7.0)	0.39
Lower limb ischemia	1 (3.0)	1 (1.8)	0.60
Pericardial effusion	4 (12.1)	11 (19.3)	0.28
Cardiac ultrasound data			
Left ventricular end-diastolic diameter ($\overline{x}$±s, cm)	5.0±0.5	5.0±0.5	0.85
Left ventricular ejection fraction ($\overline{x}$±s, %)	57.5±5.1	56.8±5.5	0.38
Aortic valve regurgitation ≥3+ (cases, %)	4 (12.1)	8 (14.0)	0.53
Location of aortic dissection rupture (cases, %)			
Ascending aorta	12 (36.4)	30 (52.6)	0.10
Arch (including arch descending isthmus)	14 (42.4)	22 (38.6)	0.45
Descending aorta	7 (21.2)	5 (8.8)	0.09

### Intraoperative data

There was no statistically significant difference between the two groups in terms of intraoperative conditions including operation time, extracorporeal circulation time, aortic block time, and deep hypothermia stoppage time (*P* > 0.05).The difference was statistically significant between the Fontus group and the Cronus group in terms of the operation of the aortic arch and root. See Table 2.

**Table 2:** Comparison of intraoperative clinical data of patients in Fontus group and Cronus group

Sports event	Fontus group (*n*=33)	Cronus group (*n*=57)	*P*-value
Surgical time (‾x±s, min)	431.1±93.4	424.6±97.9	0.76
Extracorporeal circulation time ($\overline{x}$±s, min)	209.5±52.1	205.7±49.6	0.74
Aortic block time ($\overline{x}$±s, min)	154.1±47.8	152.1±39.7	0.83
Deep cryogenic stop cycle time ($\overline{x}$±s, min)	30.8±8.4	30.6±8.6	0.69
Surgical procedures (cases, %)			
Total Arch Replacement + Stented Elephant Trunk	27 (81.8%)	24 (42.1%)	<0.01
Modified arch island anastomosis	6 (18.2%)	33 (57.9%)	<0,01
Procedures involving the root			
The bentall technique	10 (30.3)	12 (21.1)	0.23
Wheats	3 (9.1)	0 (0.0)	<0.05
Concurrent surgery			
Coronary bypass operation	0 (0.0)	2 (3.5)	0.40

### Perioperative data

There were no perioperative deaths in the whole group. The perioperative clinical outcomes of the two groups, including the comparison of the two groups in terms of mortality and postoperative renal failure, pulmonary complications, central nervous system complications, cardiac arrhythmia, reopening of the chest, as well as postoperative 24 h thoracic drainage, postoperative blood transfusion, postoperative respiratory time, postoperative ICU time, and the number of days of hospitalization, the differences were not statistically significant (*P* > 0.05). See Table 3.

**Table 3:** Comparison of perioperative and postoperative clinical data between Fontus and Cronus groups

Sports event	Fontus group (*n*=33)	Cronus group (*n*=57)	*P*-value
Perioperative mortality (cases, %)	0 (0.0)	0 (0.0)	0.99
Renal failure (cases, %)	3 (9.1)	4 (7.0)	0.51
Bedside hemodialysis (cases, %)	2 (6.1)	4 (7.0)	0.61
Pulmonary complications (cases, %)	1 (3.0)	5 (8.8)	0.28
Central nervous system dysfunction (cases, %)			
Cerebral hemorrhage	1 (3.0)	5 (8.8)	0.28
Cognitive impairment or delirium	1 (3.9)	1 (1.8)	0.60
Limb movement disorders (cases, %)	2 (6.1)	3 (5.3)	0.61
Arrhythmia (cases, %)	0 (0.0)	4 (7.0)	0.15
Re-opening of the chest for debridement (cases, %)	3 (9.1)	5 (8.8)	0.62
24 h postoperative chest drainage (‾ x±s, ml)	632.4±448.7	588.0±464.0	0.66
Postoperative suspended red blood cell transfusions (‾ x±s, ml)	1675.8±820.8	1905.4±1769.3	0.49
Postoperative plasma transfusion (‾ x±s, ml)	1030.3±521.0	1132.6±897.0	0.55
Postoperative cold-precipitated blood transfusions (‾ x±s, U)	11.8±4.5	11.8±4.5	0.65
Postoperative platelet transfusions (‾ x±s, U)	1.4±0.5	1.6±1.0	0.34
Postoperative ventilator time (‾ x±s, h)	44.0±43.6	48.5±71.3	0.74
Postoperative intensive care unit time (‾ x±s, d)	6.1±5.3	6.6±5.5	0.68
Days of hospitalization (‾ x±s, d)	21.2±17.0	21.9±12.2	0.83

*Limb movement disorders: Referring to motor dysfunction due to spinal cord ischemia

### Postoperative follow-up data

Patients completed follow-up, which ranged from 3 to 17 months, with a mean follow-up time of (7.8 ± 4.2) months. During the follow-up period, 1 case in Fontus group died in the 2nd postoperative month due to acute renal failure. In addition, 1 case was reviewed in the 3rd postoperative month and found to have in-stent leakage and underwent thoracic aortic dissection pseudo-lumen embolization, and 1 case underwent thoracic and abdominal aortic replacement for a new coarctation in the descending aorta in the 4th postoperative month.1 case in the Cronus group died of respiratory failure at 1 year after the operation. In addition, 1 case underwent endoluminal isolation of the descending aorta with a laminar stent for new entrapment of the descending aorta. The remaining patients had no neurologic or aortic complications during follow-up. No stent implantation of false lumen, branch stent displacement, branch stent distortion, or branch stent occlusion were detected on CT examination in the two groups, but the Fontus group had a significantly higher rate of thoracic descending aortic false lumen closure than the Cronus group (90.1% versus 75.4%, *P* < 0.05). After subgroup analysis of the surgical approaches in the two groups, it was found that patients undergoing island anastomosis with modified arches with the Fontus stent demonstrated a tendency to have a lower rate of postoperative endoleak than patients with the Cronus stent (0.0% versus 39.4%, *P* = 0.07). See Tables 4 and 5.

**Table 4:** Comparison of follow-up data between Fontus and Cronus groups

Sports event	Fontus group (*n*=33)	Cronus group (*n*=57)	*P*-value
Follow-up mortality (cases, %)	1 (3.0%)	1 (1.8%)	0.47
Secondary interventions (cases, %)	2 (6.1%)	1 (1.8%)	0.26
Complete closure of the distal false lumen of the stent at follow-up (cases, %)			
Level of pulmonary artery bifurcation	30 (90.1%)	43 (75.4%)	<0.05
Diaphragm level	19 (57.6%)	28 (49.1%)	0.13
Stent internal leakage (cases, %)	1 (3.0%)	13 (22.8%)	<0.01

**Table 5:** Follow-up CTA analysis of total arch Replacement + Stented elephant trunk with modified arch Island anastomosis in Fontus and Cronus groups

Sports event	Fontus & SUN’s group (*n*=27)	Cronus & SUN’s group (*n*=24)	*P*-value	Fontus & insular group (*n*=6)	Cronus & insular group (*n*=33)	*P*-value
Complete closure of the false lumen at the level of the pulmonary artery bifurcation distal to the stent (cases, %)	24 (88.9%)	21 (87.5%)	0.33	6 (100%)	22 (66.7%)	0.12
Stent internal leakage (cases, %)	1 (3.7%)	0 (0.0%)	0.37	0 (0.0%)	13 (39.4%)	0.07

## Discussion

**Fig. 1:**
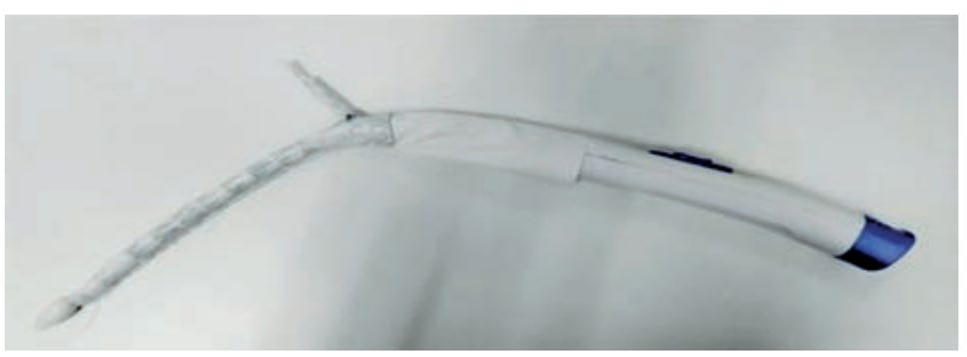
The Fontus stent prior to graft

**Fig. 2:**
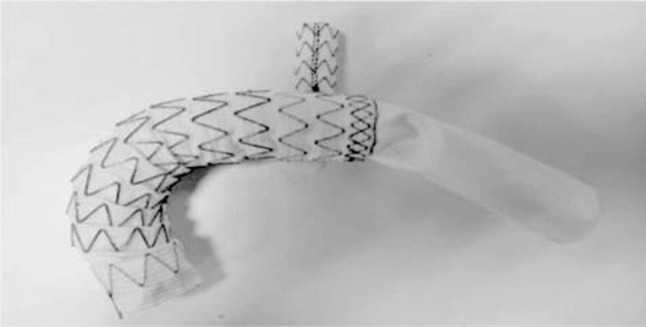
The grafted Fontus stent

Currently, arch reconstruction for Stanford type A aortic dissection involving the aortic arch in China mainly includes total aortic arch replacement and stenting with elephant trunk surgery (Total Arch Replacement + Stented Elephant Trunk), island anastomosis (en bloc technique), intra-operative open stenting, and triple-branched overlay stenting [5]. The Total Arch Replacement + Stented Elephant Trunk is used to reconstruct the aortic arch. Total Arch Replacement + Stented Elephant Trunk can reconstruct the three branches of the aortic arch and is suitable for aortic dissection involving the branches of the aortic arch, but there are problems such as high surgical difficulty, difficulty in free anastomosis of the left subclavian artery, and easy to distort and occlude the branching artificial blood vessels, etc. The islet anastomosis technique preserves the autogenous three branches of the aortic arch and avoids the problem of the distortion of the artificial blood vessels of the Total Arch Replacement + Stented Elephant Trunk, but the difficulty of anastomosis of the posterior wall of the aortic arch is high and easy to bleed. The problem with intraoperative open stents is that they are not suitable for aortic dissection where the rupture is located in the aortic arch and the head-arm trunk, and the stent is not sufficiently supportive, so that the aortic arch is susceptible to persistent endoleak of type Ib, which leads to persistent dilatation of the aortic arch; the triple-branched coated stent, on the other hand, is not suitable for use in aortic dissection because of the large variability of the anatomical structure of the branch arteries of the aortic arch, with a wide range of branch artery diameters and locations. The three-branch laminating stent, on the other hand, due to the great variability in the anatomical structure of the branch arteries of the aortic arch, the diameter and location of the branch arteries vary greatly, and the uniform type of branch vessels can not be applied to all the morphologies of the aortic arch. In view of the above problems, there is an urgent need to develop a new generation of intraoperative revascularization system to avoid some of the problems of the above arch reconstruction methods. The Fontus intraoperative single-branch stent, as an upgraded product of the Cronus stent, is designed to further reduce the difficulty of the operation and reduce the number of complications, and has the following advantages compared with the Cronus stent: the single-branch stent can avoid the most difficult and time-consuming anastomosis of the left subclavian artery, reduce the number of anastomoses, and shift the distal aortic anastomosis anteriorly (typically to Zone 2), which may reduce operative difficulty and improve safety [although this study did not show statistical differences in operative time, transfusion volume, etc., potentially related to the center’s extensive experience and the relatively small sample size]; the single-branch stent can easily adapt to the aortic arch morphology and avoid twisting of the branching stent; the stent has sufficient support at the distal end, allowing for a better rate of distal false lumen closure and avoiding type Ib endoleak. See Figs. 1 and 2.

This study compared the Fontus stent with the Cronus stent primarily because the Cronus stent was the previously widely used technology in our center, facilitating the evaluation of the new device’s benefits within our institutional framework. Compared to other commonly used FET devices internationally (e.g., Thoraflex Hybrid, E-vita Open Plus), the early outcomes of the Fontus stent appear comparable in terms of mortality and major complications [6–9], but its long-term effects and value in broader patient populations require further validation. Currently, the Fontus stent is primarily used in the Chinese market, with limited international experience and reported data.

In this study, the branch stents in the postoperative review of the aortic CTA of patients treated with Fontus stent were all in place, patent, and did not have abnormalities such as twisting, and the aortic morphology was remodeled well, and the aortic false lumen closure rate of the aortic stent-covered segments was 100%, the aortic false lumen closure rate of the aortic false lumen at the level of the stent distal to the pulmonary artery bifurcation accounted for 90.1%, the aortic false lumen closure rate of the aortic false lumen at the level of the stent distal to the diaphragm accounted for 57.6%, and was effective in improving aortic dissection both in the Sun’s procedure group and in the modified arch island anastomosis group, so the use of Fontus stent is effective in treating ATAAD. In terms of safety considerations, the postoperative morbidity and mortality rates of patients treated with Fontus stent and Cronus stent in this study were low and not statistically significant, similar to results reported for other arch surgical techniques domestically and internationally [4, 10–12]. In this study, before using Fontus stenting, the operators selected branch stents with slightly larger sizes (typically 0–10% oversizing) based on the diameter of the left subclavian artery in the patients’ preoperative aortic CTA, aiming to ensure patency while reducing the occurrence of endoleak. Postoperative review of aortic CTAs did not detect left subclavian artery occlusion or branch stent endoleak in patients treated with the Fontus stent. However, the long-term patency of the branch stent and potential endoleak risks, especially in younger patients, still require longer-term follow-up observation.

**Fig. 3:**
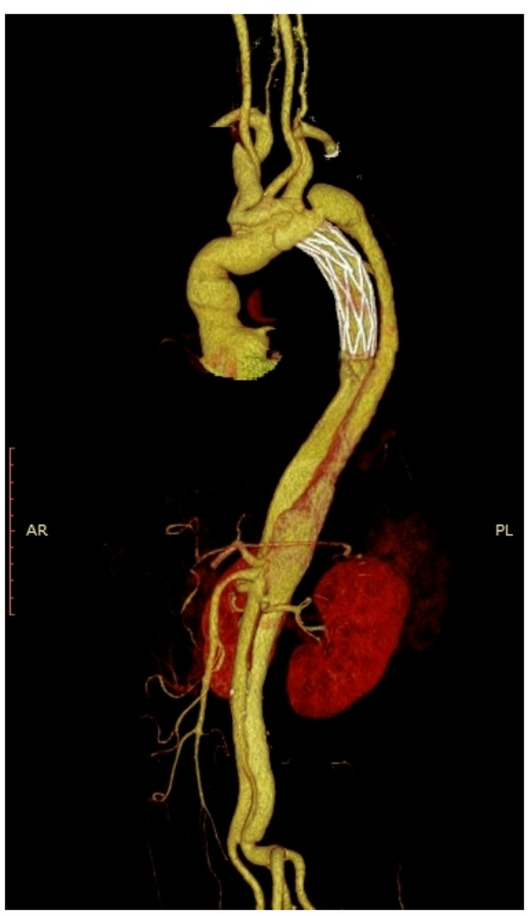
The results of the computed tomography angiography (CTA) follow-up after 3 months of Cronus stent implantation in the aorta

**Fig. 4:**
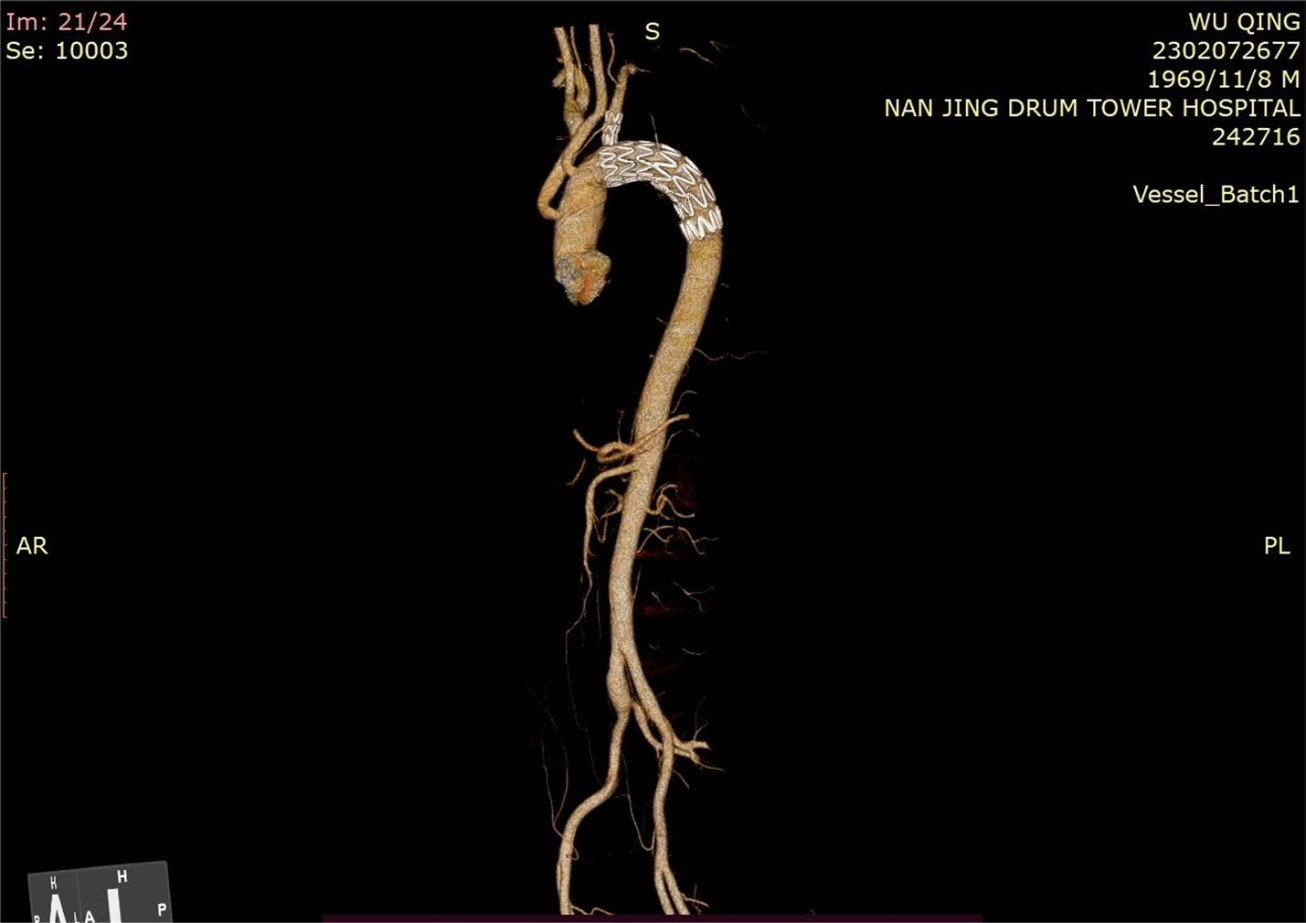
The results of the computed tomography angiography (CTA) follow-up after 3 months of Fontus stent implantation in the aorta

Furthermore, this study found that the Fontus branch-type intraoperative stent performed particularly well in the modified arch island anastomosis technique, with a postoperative stent endoleak rate of 0% in the modified arch-islander anastomosis group of patients applying the Fontus stent in the follow-up aortic CTA and a false lumen closure rate at the level of the stent distal to the pulmonary artery bifurcation of 88.9%, which both reduced the chance of postoperative stent endoleak and increased the stent The closure rate of the distal false lumen of the stent was 88.9%. The modified arch islet anastomosis technique inserts the stent into the true lumen of the descending aorta as a frozen elephant trunk, and the proximal trimmed vascular graft is sutured from the medial side of the aortic arch to cover the entire aortic arch, which avoids the risk of posterior wall anastomotic hemorrhage that is likely to occur with the conventional islet anastomosis because the entire procedure is performed within the aortic arch without resecting or extensively isolating the aortic arch. However, because the modified arch island anastomosis technique needs to be sutured at the lower edge of the left subclavian artery opening when pulling the graft back to the aortic arch, the left subclavian artery is often deeper, and it is still difficult to expose and suture, and endoleakage is still prone to occur here; whereas, the stent in the Fontus procedure shifts the anastomotic opening of the distal aorta anteriorly and releases the left subclavian artery by branching it out, which allows for a completely wall-permeable anastomosis to be performed and avoids endoleakage. anastomosis technique has a good performance. See Figs. 3 and 4.

## Conclusion

In summary, the Fontus Branch Intraoperative Stent System, as an upgraded product of the Cronus stent, offers structural and operational advantages that may flatten the learning curve and enhance safety in aortic dissection surgery, especially showing significant efficacy in the modified arch island anastomosis technique. It deserves wider application and may lead to better patient prognosis, and can be safely and effectively used in the treatment of acute Stanford type A aortic dissection. However, more multicenter, large-sample randomized controlled trials and long-term follow-up are needed to further understand its long-term therapeutic effects.

## Data Availability

The published article contains all the data that was generated and analyzed. If anyone requests the data, they can contact the corresponding author whose contact information is provided on the title page of the article.

## Abbreviations

ATAAD: Acute type a aortic dissection
